# Supplementary material for: Multi‐dataset identification of innovative feature genes and molecular mechanisms in keratoconus
Source: J Cell Mol Med. 2024 Sep 19;28(18):e70079. doi: 10.1111/jcmm.70079 (PMC11412914; doi:10.1111/jcmm.70079)
Supplement: Supplementary file 2 — Material S2. [file JCMM-28-e70079-s002.zip › 7.GSEA analysis/2.ATOH7/KEGG_GLIOMA.html]

Details for gene set KEGG\_GLIOMA[GSEA]

|  || Dataset | ATOH7.group.cls#h\_versus\_l |
| Phenotype | group.cls#h\_versus\_l |
| Upregulated in class | l |
| GeneSet | KEGG\_GLIOMA |
| Enrichment Score (ES) | -0.23342915 |
| Normalized Enrichment Score (NES) | -0.9849504 |
| Nominal p-value | 0.472 |
| FDR q-value | 1.0 |
| FWER p-Value | 1.0 |
Table: GSEA Results Summary

  

Fig 1: Enrichment plot: KEGG\_GLIOMA      
 Profile of the Running ES Score & Positions of GeneSet Members on the Rank Ordered List

  

| SYMBOL | TITLE | RANK IN GENE LIST | RANK METRIC SCORE | RUNNING ES | CORE ENRICHMENT || 1 | AKT3 | na | 9 | 1.043 | 0.1078 | No |
| 2 | SHC2 | na | 290 | 0.628 | 0.1647 | No |
| 3 | PRKCB | na | 2281 | 0.324 | 0.1409 | No |
| 4 | CAMK2D | na | 2442 | 0.313 | 0.1687 | No |
| 5 | CALML6 | na | 3497 | 0.256 | 0.1648 | No |
| 6 | PDGFB | na | 3642 | 0.250 | 0.1865 | No |
| 7 | MAP2K1 | na | 4240 | 0.228 | 0.1928 | No |
| 8 | CDKN1A | na | 5247 | 0.193 | 0.1838 | No |
| 9 | PIK3R3 | na | 6166 | 0.166 | 0.1745 | No |
| 10 | CDK6 | na | 7568 | 0.129 | 0.1475 | No |
| 11 | NRAS | na | 7693 | 0.126 | 0.1569 | No |
| 12 | PRKCA | na | 7938 | 0.120 | 0.1623 | No |
| 13 | TGFA | na | 9931 | 0.083 | 0.1133 | No |
| 14 | CDK4 | na | 9992 | 0.082 | 0.1200 | No |
| 15 | CALM3 | na | 10070 | 0.080 | 0.1261 | No |
| 16 | ARAF | na | 10150 | 0.079 | 0.1320 | No |
| 17 | GRB2 | na | 10441 | 0.075 | 0.1314 | No |
| 18 | SHC4 | na | 10680 | 0.071 | 0.1318 | No |
| 19 | EGFR | na | 12485 | 0.046 | 0.0846 | No |
| 20 | CDKN2A | na | 13514 | 0.035 | 0.0586 | No |
| 21 | EGF | na | 13899 | 0.031 | 0.0508 | No |
| 22 | MAPK1 | na | 14536 | 0.025 | 0.0350 | No |
| 23 | HRAS | na | 14559 | 0.025 | 0.0370 | No |
| 24 | TP53 | na | 14698 | 0.024 | 0.0355 | No |
| 25 | SHC1 | na | 15160 | 0.020 | 0.0242 | No |
| 26 | RAF1 | na | 15219 | 0.019 | 0.0245 | No |
| 27 | AKT1 | na | 15787 | 0.014 | 0.0097 | No |
| 28 | PIK3CD | na | 16202 | 0.011 | -0.0011 | No |
| 29 | CALM1 | na | 16251 | 0.011 | -0.0014 | No |
| 30 | MAPK3 | na | 16821 | 0.006 | -0.0171 | No |
| 31 | E2F3 | na | 17541 | -0.000 | -0.0379 | No |
| 32 | E2F1 | na | 18613 | -0.010 | -0.0678 | No |
| 33 | CCND1 | na | 20546 | -0.027 | -0.1207 | No |
| 34 | CALML3 | na | 20596 | -0.028 | -0.1192 | No |
| 35 | MAP2K2 | na | 20653 | -0.028 | -0.1180 | No |
| 36 | PIK3CB | na | 20685 | -0.028 | -0.1159 | No |
| 37 | PIK3CA | na | 21359 | -0.036 | -0.1316 | No |
| 38 | CALM2 | na | 21949 | -0.042 | -0.1442 | No |
| 39 | PIK3R2 | na | 22209 | -0.045 | -0.1470 | No |
| 40 | PDGFA | na | 22257 | -0.046 | -0.1437 | No |
| 41 | CAMK2A | na | 22272 | -0.046 | -0.1393 | No |
| 42 | SOS2 | na | 22550 | -0.049 | -0.1423 | No |
| 43 | RB1 | na | 22702 | -0.051 | -0.1414 | No |
| 44 | SHC3 | na | 22782 | -0.052 | -0.1383 | No |
| 45 | CAMK2G | na | 23214 | -0.058 | -0.1447 | No |
| 46 | PTEN | na | 23399 | -0.060 | -0.1438 | No |
| 47 | MDM2 | na | 24258 | -0.073 | -0.1609 | No |
| 48 | PLCG2 | na | 24577 | -0.078 | -0.1620 | No |
| 49 | BRAF | na | 25048 | -0.086 | -0.1666 | No |
| 50 | KRAS | na | 25083 | -0.087 | -0.1587 | No |
| 51 | MTOR | na | 25211 | -0.089 | -0.1531 | No |
| 52 | PLCG1 | na | 25238 | -0.090 | -0.1446 | No |
| 53 | PIK3CG | na | 26210 | -0.109 | -0.1613 | No |
| 54 | SOS1 | na | 26852 | -0.122 | -0.1672 | No |
| 55 | AKT2 | na | 27111 | -0.128 | -0.1614 | No |
| 56 | IGF1R | na | 27632 | -0.142 | -0.1617 | No |
| 57 | PIK3R1 | na | 27720 | -0.144 | -0.1494 | No |
| 58 | PRKCG | na | 28978 | -0.178 | -0.1672 | No |
| 59 | CALML5 | na | 29931 | -0.208 | -0.1731 | No |
| 60 | E2F2 | na | 30000 | -0.211 | -0.1532 | No |
| 61 | PDGFRB | na | 32781 | -0.349 | -0.1973 | Yes |
| 62 | IGF1 | na | 33039 | -0.370 | -0.1664 | Yes |
| 63 | PDGFRA | na | 33905 | -0.477 | -0.1420 | Yes |
| 64 | PIK3R5 | na | 34383 | -0.611 | -0.0924 | Yes |
| 65 | CAMK2B | na | 34693 | -0.989 | 0.0010 | Yes |
Table: GSEA details [plain text format]

  

Fig 2: KEGG\_GLIOMA      
 Blue-Pink O' Gram in the Space of the Analyzed GeneSet

  

Fig 3: KEGG\_GLIOMA: Random ES distribution      
 Gene set null distribution of ES for **KEGG\_GLIOMA**
